# Using Symptom and Interference Questionnaires to Identify Recovery Among Children With Anxiety Disorders

**DOI:** 10.1037/pas0000375

**Published:** 2016-08-15

**Authors:** Rachel Evans, Kerstin Thirlwall, Peter Cooper, Cathy Creswell

**Affiliations:** 1 School of Psychology and Clinical Language Sciences, University of Reading; 2 School of Psychology and Clinical Language Sciences, University of Reading and Department of Psychology, Stellenbosch University; 3 School of Psychology and Clinical Language Sciences, University of Reading

**Keywords:** anxiety disorders, child, diagnosis, parent, questionnaire

## Abstract

Questionnaires are widely used in routine clinical practice to assess treatment outcomes for children with anxiety disorders. This study was conducted to determine whether 2 widely used child and parent report questionnaires of child anxiety symptoms and interference (Spence Child Anxiety Scale [SCAS-C/P] and Child Anxiety Impact Scale [CAIS-C/P]) accurately identify recovery from common child anxiety disorder diagnoses as measured by a ‘gold-standard’ diagnostic interview. Three hundred thirty-seven children (7–12 years, 51% female) and their parents completed the ADIS-IV-C/P diagnostic interview and questionnaire measures (SCAS-C/P and CAIS-C/P) before (Time 1) and after (Time 2) treatment or wait-list. Time 2 parent reported interference (CAIS-P) was found to be a good predictor of absence of any diagnoses (area under the curve [AUC] = .81). In terms of specific diagnoses, Time 2 SCAS-C/P separation anxiety subscale (SCAS-C/P-SA) identified recovery from separation anxiety disorder well (SCAS-C-SA, AUC = .80; SCAS-P-SA, AUC = .82) as did the CAIS-P (AUC = .79). The CAIS-P also successfully identified recovery from social phobia (AUC = .78) and generalized anxiety disorder (AUC = .76). These AUC values were supported by moderate to good sensitivity (.70–.78) and specificity (.70–.73) at the best identified cut-off scores. None of the measures successfully identified recovery from specific phobia. The results suggest that questionnaire measures, particularly the CAIS-P, can be used to identify whether children have recovered from common anxiety disorders, with the exception of specific phobias. Cut-off scores have been identified that can guide the use of routine outcome measures in clinical practice.

Anxiety disorders are common in children (Cartwright-Hatton, McNicol, & Doubleday, 2006; Costello, Egger, Copeland, Erkanli, & Angold, 2011) and are associated with impairments in social, family, and school domains (Benjamin, Costello, & Warren, 1990; Ezpeleta, Keeler, Erkanli, Costello, & Angold, 2001; Strauss, Frame, & Forehand, 1987). The ‘gold-standard’ assessment tool for the diagnosis of anxiety disorders in children is the Anxiety Disorders Interview Schedule for *DSM–IV* for Children- Child and Parent Versions (ADIS-IV-C/P; Silverman & Albano, 1996). The ADIS-IV-C/P is a semistructured interview used to assess for *Diagnostic and Statistical Manual of Mental Disorders*, fourth edition (*DSM–IV*; American Psychiatric Association; APA, 1994) anxiety disorder diagnoses, on the basis of both child and parent report, and is the most commonly used tool to evaluate outcomes in treatment trials for childhood anxiety disorders (Ginsburg et al., 2011; Hudson et al., 2014). However, the ADIS-IV-C/P requires trained clinicians and considerable time to administer (averaging 134 min where children are clinically anxious; Lyneham & Rapee, 2005). It is therefore common for self- and parent-report questionnaires to be used in routine clinical practice (Children and Young People’s Improving Access to Psychological Therapies [CYP-IAPT], 2011). Self-report measures have the advantage of being relatively quick to deliver and do not require trained clinicians to administer (Simon & Bögels, 2009). However, the extent to which outcomes as assessed by these child and parent report measures of child anxiety reflect recovery as assessed by semistructured diagnostic interviews is unknown.

Receiver operating characteristic (ROC; Swets, 1988; Zweig & Campbell, 1993) methods can assess the accuracy of measures at identifying diagnoses according to established gold-standard diagnostic tools. As well as generating a score indicating a measure’s overall accuracy at identifying diagnoses (the area under the curve [AUC]), ROC can be used to identify optimum cut-off points for a measure. At each cut-off point for a given measure and diagnosis, the proportion of ‘true positive’ (sensitivity) and ‘true negative’ (specificity) individuals can be calculated. Previous recommendations for the field of child anxiety disorders have highlighted both the potential value of ROC for evaluating measures and the lack of research utilizing this method (Silverman & Ollendick, 2005).

Where ROC has been used, this has primarily been for screening purposes rather than assessing response to treatment for anxiety disorders. For example, parent and/or child report questionnaires have been identified as moderate to good screening tools for ADIS-IV-C/P social phobia and separation anxiety disorder diagnoses in children on the basis of ROC methods (Bailey, Chavira, Stein, & Stein, 2006; Villabø, Gere, Torgersen, March, & Kendall, 2012). However, the presence of any anxiety disorder has been found to be identified poorly by child reported anxiety symptoms (using the Multidimensional Anxiety Scale for Children; MASC; March, Parker, Sullivan, Stallings, & Conners, 1997) although mother report on this measure was slightly superior and therefore considered a fair measure (Villabø et al., 2012).

Although there has been some consideration of the utility of child anxiety questionnaires as screening tools, there has been little examination of their ability to identify recovery. Nonetheless self- and parent-report measures are frequently used to assess treatment outcomes (CYP-IAPT, 2011). It is clearly important that guidance is provided for clinicians on the accuracy of commonly used questionnaires at identifying recovery from anxiety disorder diagnoses including cut-offs with good sensitivity and specificity. The utility of ROC for this purpose has been demonstrated in an evaluation of the clinician-rated Pediatric Anxiety Rating Scale (PARS; Research Units on Pediatric Psychopharmacology Anxiety Study Group, 2002) as a measure of recovery from anxiety disorders in children (Caporino et al., 2013).

Given the widespread use of child and parent report questionnaires to assess treatment outcomes in clinical practice, we set out to establish the utility of two commonly used measures (Spence Child Anxiety Scale; SCAS-C/P; Nauta et al., 2004; Spence, 1998; Child Anxiety Impact Scale; CAIS-C/P; Langley, Bergman, McCracken, Piacentini, 2004; Langley et al., 2014) to identify recovery from anxiety disorders on the basis of the ADIS-IV-C/P using data from two treatment trials for child anxiety (Creswell et al., 2015; Thirlwall et al., 2013).

The SCAS-C/P was selected because this is a widely used measure of child anxiety symptoms (in its own right and in an adapted form in the Revised Child Anxiety and Depression Scale; Chorpita, Yim, Moffitt, Umemoto, & Francis, 2000). Given that functional impairment is critical in distinguishing anxiety disorder diagnoses from typical fears and worries (APA, 2013), we also elected to include a questionnaire measure of interference associated with anxiety. The CAIS-C/P is a measure of the extent to which a child’s anxiety impacts on their daily functioning in school, social and home contexts. It has been used to evaluate characteristics of anxious children (Kendall et al., 2010) and to inform evaluation of the clinician-rated PARS as a measure of response to treatment for child anxiety (Caporino et al., 2013).

A further methodological consideration is related to the facts that (a) comorbidity of anxiety disorders in children is common (Waite & Creswell, 2014), and (b) the primary outcome used across trials are typically either being free of a particular anxiety disorder (e.g., social phobia), being free of the most impairing (‘primary’) anxiety disorder, or being free of *all* anxiety disorders. For these reasons we used ROC methods to establish the ability of the CAIS-C/P and the SCAS-C/P to identify recovery, following treatment or a wait-list control, on the basis of each of these three criteria, focusing on the four most common anxiety disorders in childhood (separation anxiety disorder, social phobia, generalized anxiety disorder, and specific phobia). We evaluated both child and parent measures, because both are commonly used in routine clinical practice (CYP-IAPT, 2011) and because it is possible that diagnostic outcomes as assessed by the ADIS-IV-C/P in its standardized form may align more closely with parent than child report for preadolescent children. Indeed the ADIS-C/P, as standard, involves allocating a diagnosis if criteria are met on the basis of either child or parent report and these ‘overall’ diagnoses have previously been found to be more closely associated with outcomes from parent than child interviews (Grills & Ollendick, 2003). With these considerations in mind, this study set out to examine the extent to which child and parent report on the SCAS-C/P and CAIS-C/P reflected measures of recovery as assessed by the ADIS-C/P semistructured diagnostic interview.

## Method

### Participants

Participants were 337 children aged 7 to 12 years (M = 9.72, *SD* = 1.55) and their primary caregivers (334 mothers, 3 fathers). One hundred fifty-nine of the children were recruited as part of a randomized controlled trial (RCT) comparing two guided parent-delivered cognitive–behavioral treatments (GPD-CBT) of varying intensity to a wait-list control (Thirlwall et al., 2013). The remaining 178 of the children participated in an RCT comparing child CBT (CCBT) alone versus CCBT supplemented by CBT for maternal anxiety disorder (CCBT + MCBT) or treatment focused on the mother-child interaction (CCBT + MCI) (Creswell et al., 2015). Because the aim of this study is to evaluate the accuracy of self-report measures at identifying recovery from child anxiety disorders broadly, rather than in the context of a specific intervention, children from all treatment conditions across these two trials were included. Further details of the sample are shown in Table 1. Table 2 shows the number of participants who completed each measure at each time point. Those who did and didn’t complete each measure at each time were compared on key demographic and clinical characteristics and no consistent, significant patterns were found.[Table-anchor tbl1][Table-anchor tbl2]

### Procedure

All children were referred by primary and secondary NHS/education services to Berkshire Child Anxiety Clinic, University of Reading, UK. The children in the first trial (Thirlwall et al., 2013) were assessed before (Time 1) and after (Time 2) eight-session GPD-CBT (*n* = 50), four-session GPD-CBT (*N* = 46), or a 12-week wait-list (*n* = 63). The children in the second trial (Creswell et al., 2015) were assessed before (Time 1) and after (Time 2) eight-session CCBT (*n* = 56), eight-session CCBT + MCBT (*n* = 60), or 10-session CCBT + MCI (*n* = 62). All children and primary caregivers completed the ADIS-IV-C/P at Time 1 and Time 2 in all conditions. However, not all respondents completed the CAIS-C/P and SCAS-C/P at both time points (total *n* for each measure ranged from *n* = 247–320).

### Measures

#### Anxiety Disorders Interview Schedule for *DSM–IV*-Child and Parent Versions

Children were assigned diagnoses based on the Anxiety Disorders Interview Schedule for *DSM–IV* for Children- Child and Parent Versions (ADIS-IV-C/P; Silverman & Albano, 1996). The ADIS-IV-C/P is a clinician-administered semistructured interview to assess for *DSM–IV* anxiety, mood, and externalizing disorders in children and adolescents. The reliability and validity of child and parent versions of the ADIS-IV-C/P has been established (Silverman, Saavedra, & Pina, 2001; Wood, Piacentini, Bergman, McCracken, & Barrios, 2002). In line with the *DSM–IV*, the ADIS-IV-C/P includes questions regarding both symptoms and functional impairment associated with anxiety. Clinicians assign Clinical Severity Ratings (CSR) for each anxiety disorder following ADIS-IV-C/P interviews on a scale of 0 (*complete absence of psychopathology*) to 8 (*severe psychopathology*). As is conventional and recommended in the ADIS-IV-C/P clinician manual (Albano & Silverman, 1996), children were assigned diagnoses where the CSR was 4 (moderate psychopathology) or greater on the basis of either child or parent report and the higher of the two was allocated. The anxiety disorder with the highest CSR was allocated as the primary disorder. The assessors were all psychology graduates (BSc/MSc in psychology) and were trained to administer and score the ADIS-IV-C/P through listening to audio-recorded assessments, role-plays, verbal instructions, and consensus meetings. In both trials, assessors were blind to treatment condition, and at least the first 20 interviews of each assessor were discussed with a consensus team before formal reliability checks. After assessors had achieved reliability of κ = .85, one in every six interviews was discussed with the consensus team to prevent rater drift. Interrater reliability for the team in assigning individual diagnoses at posttreatment was excellent (child report diagnosis: κ = .94–.99 parent report diagnosis: κ = .97–.98).

#### Spence Children’s Anxiety Scales

The Spence Children’s Anxiety Scales (SCAS-C/P; Nauta et al., 2004; Spence, 1998) are parent- and child-report scales comprising 38 items referring to common symptoms of child anxiety disorders. A number of subscales are formed from these 38 items. Relevant to the present study, six of the items form a separation anxiety subscale (SCAS-C/P-SA), six form a social phobia subscale (SCAS-C/P-SP), six form a generalized anxiety subscale (SCAS-C/P-GA), and five form a physical injury fears subscale (SCAS-C/P-PI) which is relevant to specific phobias. Respondents are asked to indicate how accurate each of the 38 statements are on a 4-point scale of *never*, *sometimes*, *often*, and *always*. The child and parent versions are identical apart from adjustments to make the wording suitable for each and the inclusion of ‘filler’ items in the child version. The SCAS-C/P has been widely used and established as having good concurrent validity (Whiteside & Brown, 2008), discriminant validity (Nauta et al., 2004; Spence, 1998; Whiteside & Brown, 2008), convergent validity with other commonly used measures (Essau, Muris, & Ederer, 2002; Nauta et al., 2004; Spence, 1998; Whiteside & Brown, 2008), and acceptable test–retest reliability (Spence, 1998). The good psychometric properties of the SCAS-C/P have been established in children aged 6 to 18 years (Spence, 1998; Nauta et al., 2004), and it has been widely used with children of the age range considered here (e.g., Hudson et al., 2014; Vigerland et al., 2016). In the present sample, internal consistency was good for total SCAS-C/P total scores at Time 1 and Time 2 (α = .88–.93), although was more variable for subscales (see Table 2).

#### Child Anxiety Impact Scales

The Child Anxiety Impact Scales (CAIS-C/P; Langley et al., 2004; Langley et al., 2014) are 33-item parent- and child-report measures, containing 27 items which refer to impact within three categories: school activities, social activities, and home/family activities. Four items relate to global impact. Respondents are asked to indicate on a 4-point scale the extent to which anxiety has caused trouble with each activity (i.e., ‘not at all,’ ‘just a little,’ ‘pretty much,’ or ‘very much’). Parent and child versions are identical apart from minor changes to wording. The CAIS-C/P has been shown to have good internal consistency and convergent validity with other measures of child anxiety (Langley et al., 2014). The psychometric properties of the CAIS-C/P have been established in children aged 7 to 17 years (Langley et al., 2004, 2014). Internal consistency was good for CAIS-P at Time 1 and 2 and for CAIS-C at Time 2 in the present sample (α = .70-.94) but not Time 1 (α = .52; see Table 2).

### Data Analysis

Consistent with existing literature evaluating the accuracy of measures at identifying diagnoses by the ADIS-IV-C/P (Bailey et al., 2006; Caporino et al., 2013; Villabø et al., 2012), and recommendations for this area of research (Silverman & Ollendick, 2005), ROC (Swets, 1988; Zweig & Campbell, 1993) methods were used to determine the ability of Time 2 total scores on the parent and child versions of the CAIS and SCAS, plus subscales of the SCAS, to identify recovery according to the ADIS-IV-C/P. Analyses were also conducted using change scores between Time 1 and Time 2, but no clear advantage was found of looking at these over Time 2 scores alone so we have focused our report on Time 2 scores. Furthermore, analyses were conducted separately on the two trials and just on the children who received treatment (i.e., excluding those in wait-list conditions). No clear or systematic differences were found in the results of any of these groups compared with the overall 337 children (see the Appendix for results with treated group only). Therefore, only the results of analyses with these 337 children are presented.

A number of values are generated from ROC methods. The AUC is a single global indicator of a test’s ability to correctly classify individuals’ diagnostic status, with an AUC of 1.0 indicating a perfect test and .50 indicating a test which classifies individuals at chance level. AUCs of .70 to .74 have been described variously as moderate (van Gastel & Ferdinand, 2008), fair (Villabø et al., 2012), and as representing a large effect size (Rice & Harris, 2005). The present study therefore used an AUC of .70 as the minimum value for a measure to be considered at least moderately accurate at identifying recovery from diagnoses according to ADIS-IV-C/P. For each diagnostic outcome, results of analyses using ROC methods are only presented for measures which achieved an AUC exceeding .70. MedCalc for Windows (Version 15.6) was used to establish that all ROC analyses included sufficient numbers of children with both positive and negative outcomes to achieve an AUC of .70.

ROC analyses also generate sensitivity and specificity values which, in the context of this study, indicate the ability of each questionnaire (CAIS/SCAS) to correctly identify children who have recovered from a diagnosis and children who have retained a diagnosis, respectively, on a scale of 0 to 1. In the present context, a higher cut-off score leads to greater sensitivity and lower specificity whereas a lower cut-off score results in lower sensitivity but greater specificity. To minimize the risk of children who retained diagnoses being incorrectly classified as recovered, the highest cut-off score with a specificity of at least .70 was selected for each measure.

## Results

At the Time 2 (posttreatment/wait list) assessment, 47% of children no longer had the primary diagnosis they had been assigned at Time 1, and 27% were free of all anxiety disorders. Of the children diagnosed with separation anxiety disorder at Time 1, 53% were recovered from this diagnosis at Time 2. The equivalent figures for recovery from social phobia, generalized anxiety disorder, and specific phobias at Time 2 were 37%, 60%, and 39%, respectively.

### Identifying Absence of All Anxiety Diagnoses With Time 2 SCAS-C/P and CAIS-C/P Scores

Although SCAS-C, SCAS-P and CAIS-P all achieved AUCs exceeding .70 for identifying absence of any anxiety diagnoses, both the SCAS-C and SCAS-P showed relatively poor sensitivity at their respective cut-off scores of 22 and 18. However, the CAIS-P achieved an AUC of .81 which, along with acceptable sensitivity (.70) and specificity (.70) values, indicates that a CAIS-P score of below 7 is a good indicator that a child has no anxiety diagnoses as determined by the ADIS-IV-C/P (see Table 3).[Table-anchor tbl3]

### Identifying Recovery From Primary Diagnosis With Time 2 SCAS-C/P and CAIS-C/P Scores

As shown in Table 3, the only measure to achieve an AUC exceeding .70 for identifying loss of primary diagnosis was the CAIS-P at a cut-off score of 8. However, the relatively poor sensitivity at this cut-off (.61) limits the extent to which it can be considered useful for this outcome as it indicates that a considerable proportion who recovered from their primary diagnosis actually had CAIS-P scores exceeding 8.

### Identifying Recovery From Particular Anxiety Diagnoses With Time 2 SCAS-C/P and CAIS-C/P Scores

As shown in Table 3, recovery from separation anxiety disorder was successfully identified by both child (AUC = .80) and parent (AUC = .82) reports on the SCAS separation anxiety subscale. The (total) CAIS-P score was only marginally less successful (AUC = .79) at a cut-off point of 12. All three of these measures also achieved acceptable sensitivity (.70–.76) and specificity (.71–.73) at their respective cut-off scores, meaning they can be considered as moderate to good at identifying recovery from separation anxiety disorder. Although both SCAS-C and SCAS-P total scores achieved moderate AUC values for identifying loss of separation anxiety disorder, the relatively poor sensitivity at their cut-off scores (.61 and .62, respectively) limits the extent to which they can be considered useful for assessing this outcome.

Recovery from social phobia was identified well by CAIS-P (AUC = .78) with good sensitivity (.78) and acceptable specificity (.71) at a cut-off score of 13. However, both SCAS-P total and social phobia subscales had poor sensitivity (.55 and .56, respectively) at their respective cut-off scores.

The only measure to achieve an AUC exceeding .70 for identifying recovery from generalized anxiety disorder was the CAIS-P (AUC = .76), at a cut-off score of 12. This was associated with acceptable sensitivity (.70) and specificity (.72), suggesting that this measure is useful for identifying recovery from generalized anxiety disorder.

Although child and parent reports on the SCAS physical injury subscale achieved moderate AUC values for identifying recovery from specific phobias, the poor sensitivity (.46 and .48, respectively) at a cut-off score of 2.5 prevents the conclusion that either accurately identify this outcome. No other measure accurately identified recovery from specific phobias.

## Discussion

The parent-report CAIS score successfully identified absence of any anxiety diagnosis as established using the standard administration of the ADIS-C/P, albeit with a low cut-off score. Although no measure was particularly successful at identifying whether children recovered from their primary diagnosis, at least one of the questionnaire measures successfully identified recovery from each of the specific diagnoses considered here (apart from specific phobia). In general parent-report scores tended to be better at identifying diagnostic outcomes from the ADIS-C/P than child scores, with the CAIS-P performing best.

There is a paucity of research using ROC methods to evaluate self-report measures as indices of diagnostic outcomes, limiting comparison with other findings. The superior AUC, sensitivity, and specificity values found for the PARS at identifying recovery according to ADIS-IV-C/P (Caporino et al., 2013) are understandable given that they are both clinician-rated measures. However, the present findings demonstrate that questionnaire measures can be used as acceptable indicators of some ADIS-C/P diagnostic outcomes on the basis of having AUC values of at least 0.70 and acceptable sensitivity and specificity. Specifically, based on the data sets used here, cut off-scores of the CAIS-P and separation anxiety subscale of the SCAS-C/P can be recommended to assess recovery for children with separation anxiety disorder (CAIS-P < 12; SCAS-C-SA <6; SCAS-P-SA <7), with CAIS-P cut-off scores recommended to assess recovery from social phobia (CAIS-P < 13) and generalized anxiety disorder (CAIS-P < 12). Notably, although the separation anxiety subscale of the SCAS-C/P performed well at identifying recovery from separation anxiety disorder, the social phobia subscale of the SCAS-C/P was not a good indicator of recovery from social phobia and nor was the generalized anxiety subscale a good indicator of recovery from generalized anxiety disorder. Furthermore, neither the SCAS-C/P (total or physical injury subscale) nor the CAIS-C/P was accurate at identifying recovery from specific phobias. This may well be accounted for by the fact that impairment relates to restricted fear domains and contexts and more closely tailored measures may be required, which is also likely to account for the relatively low internal consistency of the SCAS specific phobia scale.

The superior performance of the CAIS over the SCAS for the majority of outcomes may well reflect the fact that meeting diagnostic criteria is dependent on there being significant functional impairment (APA, 1994, 2013). It is also important to note that the CAIS-P cut-off point for identifying children with no anxiety diagnoses (i.e., complete recovery) was markedly lower than those for the individual anxiety diagnoses. This is likely to reflect the high comorbidity of anxiety disorders in children (Waite & Creswell, 2014), meaning that a child who has recovered from their primary or any specific diagnosis may well retain at least one other anxiety diagnosis.

Parent reports were generally better at identifying diagnostic outcomes on the ADIS-C/P than children’s self-reports: consistent with previous findings of superior accuracy of mother rather than child report as screening tools for child anxiety (Villabø et al., 2012). This may potentially relate to limitations associated with children’s reflective abilities, recognition of the broader interference (e.g., to family life) of their symptoms, and/or to anxious children’s tendency to ‘fake good’ (Kendall & Chansky, 1991). Although we restricted our analyses to a relatively narrow age group (7–12 years), it is likely that the utility of child report will vary substantially within this age group. We would also anticipate that quite different rates of diagnostic accuracy would be found with older populations (i.e., adolescents) and future studies are required with this age group. It is also the case that the superiority of parent report may reflect the fact that because this was a referred sample parents will have been aware of their child’s symptoms and the associated functional impairments. Whether similar findings would be found in a longitudinal community study remains unclear.

A further possibility is that the greater agreement between parent (compared with child) report questionnaire scores and diagnostic outcomes reflected the tendency for poor parent–child agreement on the ADIS (Choudhury, Pimentel, & Kendall, 2003; Comer & Kendall, 2004; Grills & Ollendick, 2003) and for diagnostic outcomes of the ADIS interviews to be more closely related to parent reports (Grills & Ollendick, 2003). We used the standard administration of the ADIS-IV-C/P, that is, diagnoses were given if either parent or child report met diagnostic criteria. As highlighted by existing literature (e.g., Grills & Ollendick, 2003; Hawley & Weisz, 2003) it is possible, or indeed likely, that the stronger performance of parent reports on questionnaires in predicting recovery in the present study reflects a tendency for final ADIS-C/P diagnoses to reflect parent reports to a greater extent than child reports. In the present study, children and parents showed moderate agreement (62% to 81% across diagnoses) although final clinician-awarded ADIS-IV-C/P diagnoses did more closely reflect parent reports (87% to 92% across diagnoses) than child reports (68% to 86% across diagnoses). This is not to say that child reports are unimportant in assessing recovery from anxiety disorders, and the results of the present study should be considered in the context of the greater parental influence on ADIS-IV-C/P outcomes when administered in its standard form with preadolescent children. Further research would benefit from the addition of more objective assessments of recovery from child anxiety disorders which are independent of child and parent reports (e.g., using behavioral observations).

A number of study limitations need to be highlighted. Despite the fact that we used established measures where good psychometric properties have previously been established, there was considerable variance in internal consistency of measures. The poor internal consistency of the physical injury subscale of the SCAS-C/P further supports our conclusion that it is not a suitable measure of recovery from specific phobias, and is likely to reflect the fact that it is comprised of items regarding discrete fears (e.g., dogs, heights). The CAIS-C also showed poor internal consistency at Time 1, although not at Time 2, and it is unclear why this was found. Nevertheless, most of the Time 2 measures that performed well in terms of identifying recovery from anxiety diagnoses also showed good to excellent internal consistency. The one exception was the separation anxiety subscale of the SCAS-P, which showed poor internal consistency despite comparing favorably with other measures in its accuracy at identifying recovery from separation anxiety disorder.

This was a relatively high socioeconomic group, predominantly of nonminority ethnicity, with children from a limited age range (7–12 years). The study also reports on the ADIS-IV-C/P which assesses *DSM–IV* diagnoses, although notably there have been few changes in relation to the particular diagnoses covered here in *DSM–5*.

These limitations notwithstanding, the current study provides evidence to support the use of the parent-report CAIS questionnaire as a valid tool to assess recovery from childhood anxiety disorders generally and from separation anxiety disorder, social phobia, and generalized anxiety disorder, specifically in 7- to 12-year-old children. Furthermore, the SCAS separation anxiety subscale child and parent reports were able to accurately identify the absence of childhood separation anxiety disorder. These findings are likely to be of value for monitoring outcomes and guiding decision making in routine clinical practice where full diagnostic interviews may not be feasible.

## Figures and Tables

**Table 1 tbl1:** Demographic Characteristics of the Sample (N = 337)

Characteristic	*N*	%
Gender		
Male	164	48.7
Female	173	51.3
Ethnicity		
White British	289	85.8
Parental marital status		
Married, remarried, living with partner	254	75.4
Single parent	74	21.4
Not stated	9	2.7
Socio-economic status of family		
Higher/Professional	192	57
Other employed	99	29.4
Unemployed	10	3
Not stated	36	10.7
Presence of common anxiety diagnoses		
Separation anxiety disorder	190	56.4
Social phobia	217	64.4
Generalized anxiety disorder	212	62.9
Specific phobia	156	46.3
Child primary diagnosis (ADIS-IV-C/P)		
Separation anxiety disorder	81	24
Social phobia	73	21.7
Generalized anxiety disorder	92	27.3
Specific phobia	62	18.5
Other anxiety disorders	29	8.7
Child primary diagnosis CSR		
Moderate (CSR 4)	31	9.2
Moderate (CSR 5)	93	27.6
Severe (CSR 6)	175	51.9
Severe (CSR 7)	36	10.7
Very severe (CSR 8)	2	.6
Presence of other psychiatric disorders (ADIS-IV-C/P)		
Dysthymia	18	5.3
MDD	28	8.3
ADHD (all types)	46	13.6
ODD	64	19
*Note.* CSR = clinical severity rating; MDD = major depressive disorder; ADHD = attention-deficit hyperactivity disorder; CD = conduct disorder; ODD = oppositional defiant disorder.		

**Table 2 tbl2:** Descriptive Statistics and Cronbach’s Alphas for Child and Parent Report Anxiety Measures at Time 1 and Time 2

	Time 1				Time 2			
Measure	*N*	*M*	*SD*	α	*N*	*M*	*SD*	α
SCAS-C total	320	39.30	18.38	.91	294	27.12	16.37	.93
SCAS-C separation anxiety subscale	323	8.65	3.94	.64	301	5.66	3.86	.78
SCAS-C social phobia subscale	320	7.48	3.92	.73	293	5.30	3.76	.71
SCAS-C generalized anxiety subscale	320	8.24	3.51	.73	294	6.12	4.50	.81
SCAS-C physical injury fears subscale	320	5.48	3.23	.51	294	4.12	2.71	.44
SCAS-P total	308	40.03	16.15	.88	247	23.56	12.30	.90
SCAS-P separation anxiety subscale	307	10.42	3.45	.52	251	6.62	3.13	.59
SCAS-P social phobia subscale	307	9.95	3.92	.74	246	7.25	3.46	.78
SCAS-P generalized anxiety subscale	308	8.74	3.45	.73	248	5.26	2.80	.76
SCAS-P physical injury fears subscale	308	5.89	3.17	.47	248	3.93	2.70	.57
CAIS-C total	319	18.34	13.90	.52	294	12.73	13.12	.94
CAIS-P total	283	19.57	14.66	.70	250	12.02	11.23	.90
*Note.* Subscale scores are given for children with relevant diagnoses at Time 1. SCAS-C = Spence Children’s Anxiety Scale (child report); SCAS-P = Spence Children’s Anxiety Scale (parent report); CAIS-C = Child Anxiety Impact Scale (child report); CAIS-P = Child Anxiety Impact Scale (parent report).								

**Table 3 tbl3:** Results of ROC Analyses for Measures Achieving Area Under the Curve of > .70 for Identifying Absence of All Anxiety Diagnoses and Recovery From Primary Diagnosis, Separation Anxiety Disorder, Social Phobia, Generalized Anxiety Disorder, and Specific Phobia

Outcome	Time 2 measure	Cut-Off score	Sensitivity	Specificity	AUC
Absence of any anxiety diagnoses	SCAS-C	22	.66	.70	.73
	SCAS-P	18	.60	.72	.75
	CAIS-P	7	.70	.70	.81
Recovery from primary diagnosis	CAIS-P	8	.61	.73	.75
Recovery from separation anxiety disorder	SCAS-C	28	.61	.70	.71
	SCAS-P	24	.62	.73	.77
	SCAS-C-SA	6	.76	.73	.80
	SCAS-P-SA	7	.70	.71	.82
	CAIS-P	12	.72	.71	.79
Recovery from social phobia	SCAS-P	21	.55	.72	.70
	SCAS-P-SP	6	.56	.74	.74
	CAIS-P	13	.78	.71	.78
Recovery from generalized anxiety disorder	CAIS-P	12	.70	.72	.76
Recovery from specific phobias	SCAS-C-PI	2.5	.46	.77	.73
	SCAS-P-PI	2.5	.48	.73	.71
*Note.* AUC = area under the curve; SCAS-C = Spence Children’s Anxiety Scale (child report); SCAS-P = Spence Children’s Anxiety Scale (parent report); CAIS-P = Child Anxiety Impact Scale (parent report); SCAS-C-SA = SCAS separation anxiety subscale (child report); SCAS-P-SA = SCAS separation anxiety subscale (parent report); SCAS-P-SP = SCAS social phobia subscale (parent report); SCAS-C-PI = SCAS physical injury fears subscale (child report); SCAS-P-PI = SCAS physical injury fears subscale (parent report).					

## AUC Values for Those Who Received Treatment and for the Overall Sample for Each Diagnostic Outcome at Time 2

**Table:**

		AUC	
Outcome at Time 2	Measure	Treated (*n* = 274)	Overall (*n* = 337)
Absence of any anxiety diagnosis	SCAS-C	.74	.73
	SCAS-P	.76	.75
	CAIS-C	.68	.69
	CAIS-P	.80	.81
Recovery from primary diagnosis	SCAS-C	.67	.67
	SCAS-P	.70	.68
	CAIS-C	.64	.64
	CAIS-P	.74	.75
Recovery from separation anxiety disorder	SCAS-C	.69	.71
	SCAS-P	.77	.77
	SCAS-C-SA	.79	.80
	SCAS-P-SA	.80	.82
	CAIS-C	.62	.64
	CAIS-P	.77	.79
Recovery from social phobia	SCAS-C	.64	.66
	SCAS-P	.69	.70
	SCAS-C-SP	.63	.64
	SCAS-P-SP	.72	.74
	CAIS-C	.63	.64
	CAIS-P	.77	.78
Recovery from generalized anxiety disorder	SCAS-C	.63	.64
	SCAS-P	.66	.63
	SCAS-C-GA	.65	.64
	SCAS-P-GA	.62	.63
	CAIS-C	.66	.66
	CAIS-P	.73	.76
Recovery from specific phobias	SCAS-C	.60	.59
	SCAS-P	.64	.65
	SCAS-C-PI	.70	.73
	SCAS-P-PI	.72	.71
	CAIS-C	.57	.56
	CAIS-P	.58	.58
*Note.* AUC = area under the curve; SCAS-C = Spence Children’s Anxiety Scale (child report); SCAS-P = Spence Children’s Anxiety Scale (parent report); CAIS-C = Child Anxiety Impact Scale (child report); CAIS-P = Child Anxiety Impact Scale (parent report); SCAS-C-SA = SCAS separation anxiety subscale (child report); SCAS-P-SA = SCAS separation anxiety subscale (parent report); SCAS-C-SP = SCAS social phobia subscale (child report); SCAS-P-SP = SCAS social phobia subscale (parent report); SCAS-C-GA = SCAS generalized anxiety subscale (child report); SCAS-P-GA = SCAS generalized anxiety subscale (parent report); SCAS-C-PI = SCAS physical injury fears subscale (child report); SCAS-P-PI = SCAS physical injury fears subscale (parent report).			
